# Population structure, genetic diversity, and selective signature of Chaka sheep revealed by whole genome sequencing

**DOI:** 10.1186/s12864-020-06925-z

**Published:** 2020-07-29

**Authors:** Jie Cheng, Huangqing Zhao, Ningbo Chen, Xiukai Cao, Quratulain Hanif, Li Pi, Linyong Hu, Buren Chaogetu, Yongzhen Huang, Xianyong Lan, Chuzhao Lei, Hong Chen

**Affiliations:** 1 Key Laboratory of Animal Genetics, Breeding and Reproduction of Shaanxi Province, College of Animal Science and Technology, Northwest A&F University, Yangling, 712100 Shaanxi China; 2 National Institute for Biotechnology and Genetic Engineering, Jhang Road, 577, Faisalabad, Pakistan; 3 Pakistan Institute of Engineering and Applied Sciences, Nilore, Islamabad Pakistan; 4 Key Laboratory of Adaptation and Evolution of Plateau Biota, Northwest Institute of Plateau Biology, Chinese Academy of Sciences, Xining, 810001 Qinghai China; 5 Animal Disease Control Center of Haixi Mongolian and Tibetan Autonomous Prefecture, Delingha, 817000 Qinghai China

**Keywords:** Chaka sheep, Population structure, Specific variation, Fst and XP-CLR

## Abstract

**Background:**

Chaka sheep, named after Chaka Salt Lake, are adapted to a harsh, highly saline environment. They are known for their high-grade meat quality and are a valuable genetic resource in China. Furthermore, the Chaka sheep breed has been designated a geographical symbol of agricultural products by the Chinese Ministry of Agriculture.

**Results:**

The genomes of 10 Chaka sheep were sequenced using next-generation sequencing, and compared to that of additional Chinese sheep breeds (Mongolian: Bayinbuluke and Tan; Tibetan: Oula sheep) to explore its population structure, genetic diversity and positive selection signatures. Principle component analysis and a neighbor-joining tree indicated that Chaka sheep significantly diverged from Bayinbuluke, Tan, and Oula sheep. Moreover, they were found to have descended from unique ancestors (K = 2 and K = 3) according to the structure analysis. The Chaka sheep genome demonstrated comparable genetic diversity from the other three breeds, as indicated by observed heterozygosity (Ho), expected heterozygosity (He), runs of homozygosity (ROH), linkage disequilibrium (LD) decay. The enrichment analysis revealed that in contrast to Mongolian or Tibetan lineage groups, the genes annotated by specific missense mutations of Chaka sheep were enriched with muscle structure development (GO:0061061) factors including insulin-like growth factor 1 (*IGF1*), growth differentiation factor 3 (*GDF3*), histone deacetylase 9 (*HDAC9*), transforming growth factor beta receptor 2 (*TGFBR2*), and calpain 3 (*CAPN3*), among others. A genome-wide scan using Fst and XP-CLR revealed a list of muscle-related genes, including neurofibromin 1 (*NF1*) and myomesin 1 (*MYOM1*), under potential selection in Chaka sheep compared with other breeds.

**Conclusions:**

The comprehensive genome-wide characterization provided the fundamental footprints for breeding and management of the Chaka sheep and confirmed that they harbor unique genetic resources.

## Background

Chaka Salt Lake, a hypersaline lake, was formed nearly 11.4 ka BP (kilo years before present) in Wulan county, Qinghai province in northwestern China. Salinity in the lake increased by 7.2 cal ka BP (calibrated kilo years before present) [1]. Chaka sheep, a natural inhabitant of the lake, have naturally adapted to the hypersaline environment and plateau habitat. Also called Qinghai plateau half-fine wool sheep for fur and meat, the breed has been designated a geographical symbol of agricultural products by the Chinese Ministry of Agriculture.

Sheep are ruminants and one of the main sources of wool, hide, and meat for humans. Chinese indigenous sheep breeds include Mongolian, Kazakh, and Tibetan lineage groups according to their geographical distribution and genetic relationships (China National Commission of Animal Genetic Resources 2011) [2]. Geographically, the Kazakh lineage is mainly concentrated in the Xinjiang area, while the Tibetan lineage (TL) group inhabits the Yunnan-Guizhou plateau. However, the Mongolian lineage (ML) has the largest population and is widely distributed in northern, central, and eastern areas of China. Among these populations, Chaka (CKA), Tan (TAN), Bayinbuluke (BYK), and Oula (OLA) sheep are widespread in Qinghai, Ningxia, Xinjiang, and Qinghai, respectively, in China (Supplementary Figure 1). TAN and BYK sheep are within the Mongolian lineage, OLA is within the Tibetan lineage, and the lineage of CKA sheep has not yet been identified.

To characterize the Chaka sheep’s genome, it could be compared to the genomes of Mongolian (TAN and BYK sheep) and Tibetan lineage sheep (OLA sheep). It is possible that artificial selection positively drives breed diversity. Most Chinese domestic sheep are reared for meat, wool, or body growth purposes while some varieties are multipurpose. TAN sheep are mainly reared for lambskin, BYK sheep are raised for mutton, and OLA sheep grow rapidly and exhibit good stress resistance. The CKA breed, known as tributary sheep in ancient times, is well-known for high mutton quality and exceptional wool production, making it an excellent multipurpose breed.

Although SNP arrays have been extensively used to identify genetic variations [3–6], the results are limited by low probe density. High-throughput sequencing has allowed for improved characterization of genome characteristics and population structure at a genome-wide level [7]. Several studies have explored the genome diversity of goat [8], cattle [9], and pigs [10] by next-generation sequencing. However, to our knowledge, a survey of the genome-wide genetic features of CKA sheep has not been conducted. To investigate genetic diversity and population structure of CKA sheep, genomes of 40 sheep, including 10 each of the CKA, TAN, BAK, and OLA breeds, were assembled to the sheep reference genome (REF). Our analyses provided new insights into the genetic diversity, population structure, and selective signature of the CKA breed compared to the other three breeds, which will improve the conservation programs for CKA sheep.

## Results

### Whole-genome sequencing and genetic variation

A total of 40 individual sheep, including CKA, TAN, BAK, and OLA (for each, *n* = 10) breeds were used to call 14.9 million autosomal SNPs and 1.54 million indels, focusing mainly on the intronic region by GATK (Supplementary Table 1). Moreover, 90,870 missense variants and 184,319 synonymous variants were found, and 1154 deletions and 791 insertions caused frameshift mutations.

Three metrics were used to estimate the genetic diversities of the sheep breeds in the current study. The range of expected heterozygosity (He) and observed heterozygosity (Ho) were 0.226–0.316 and 0.238–0.240, respectively (Supplementary Table 2). The expected heterozygosity was observed to be slightly higher than the observed heterozygosity in all populations. The expected heterozygosity of CKA sheep was much higher than that of other breeds on average. Furthermore, approximately 65% of the single nucleotide polymorphisms (SNPs) were highly variable with a minor allele frequency (MAF) > 0.3.

**Fig. 1:**
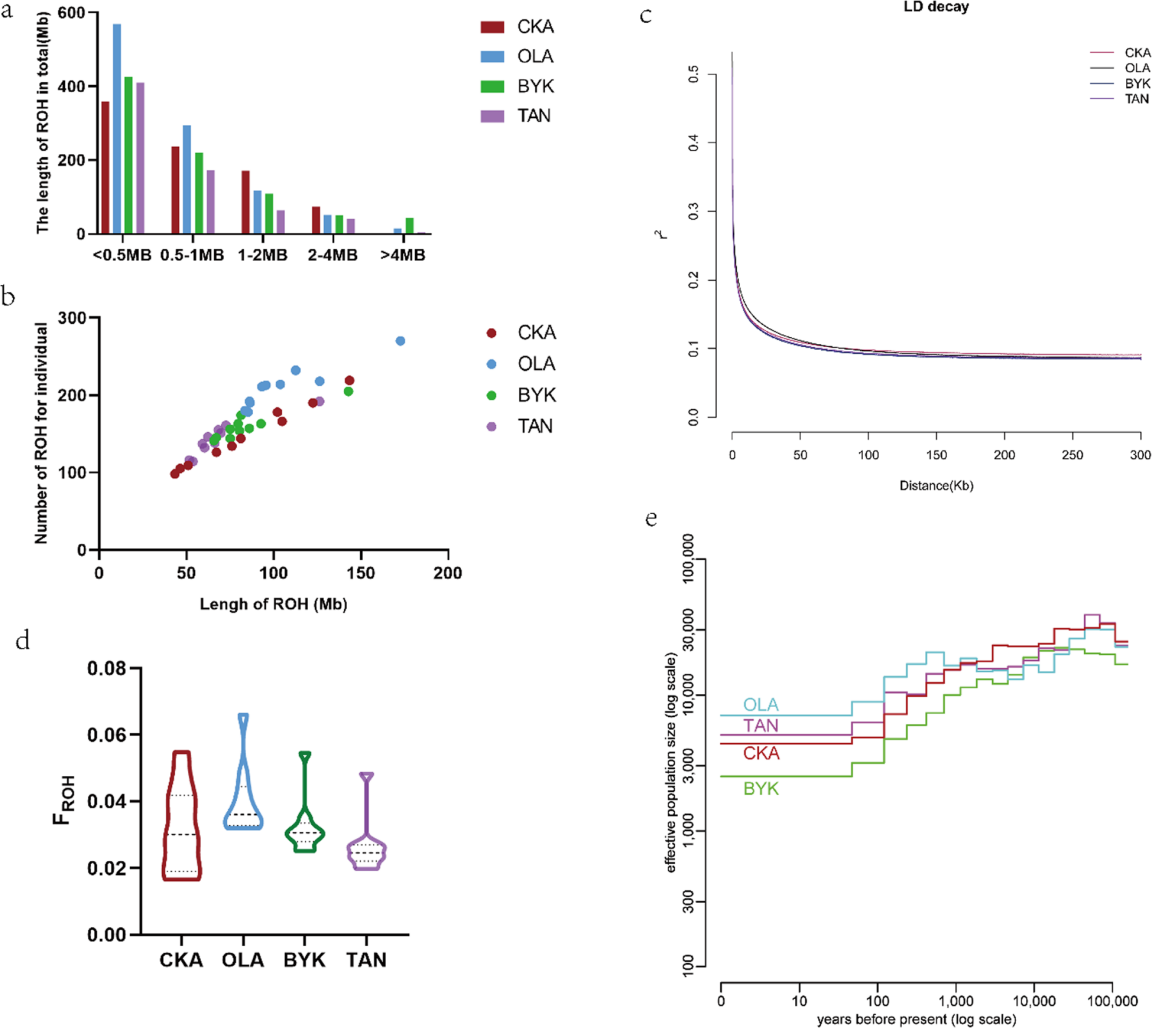
Information about ROH, inbreeding coefficient (F_ROH_), LD decay, and effective population size. **a.** The total length of ROH in different length categories. **b.** The distribution of the length and number of ROH in different individuals**. c.** Linkage disequilibrium decay (LD decay) in four breeds. **d.** Violin plot of genomic inbreeding coefficient in four sheep populations. **e.** Effective population sizes of different generations in four breeds. CKA, Chaka sheep; OLA, Oula sheep; BYK, Bayinbuluke sheep and TAN, Tan sheep

Demographic inferences in sheep population were based on the analyses of ROH, F_ROH_, LD decay, and effective population size. The ROH number varied between the sheep populations, ranging from ~ 43.37 Mb to ~ 172.76 Mb, while the average ROH lengths in CKA, BYK, OLA, and TAN sheep were approximately 83.80 Mb, 84.56 Mb, 104.60 Mb, and 69.00 Mb, respectively, suggesting the following order of breed genetic diversity: TAN > CKA > BYK > OLA (Table 1, detail in Supplementary Table 2 and Supplementary Table 3). The total length of ROH in CKA sheep, in the range of < 0.5 Mb, was lowest, suggesting CKA sheep have higher genetic diversity (Fig. 1a, b). Moreover, the result from LD decay (Fig. 1c) was nearly consistent with those from the ROH profile, in which the lowest genetic diversity was found in the OLA breed. Inbreeding coefficients (F_ROH_) among the sheep populations ranged from 0.01658–0.06606 (Table 1), while lower inbreeding coefficients were observed in TAN (F_ROH_ = 0.0264) and CKA breeds (F_ROH_ = 0.032) on average (Fig. 1d). The effective population sizes of the four breeds 1000 years before present were OLA sheep > TAN sheep > CKA sheep > BYK sheep (Fig. 1e).

### Population structure

**Fig. 2:**
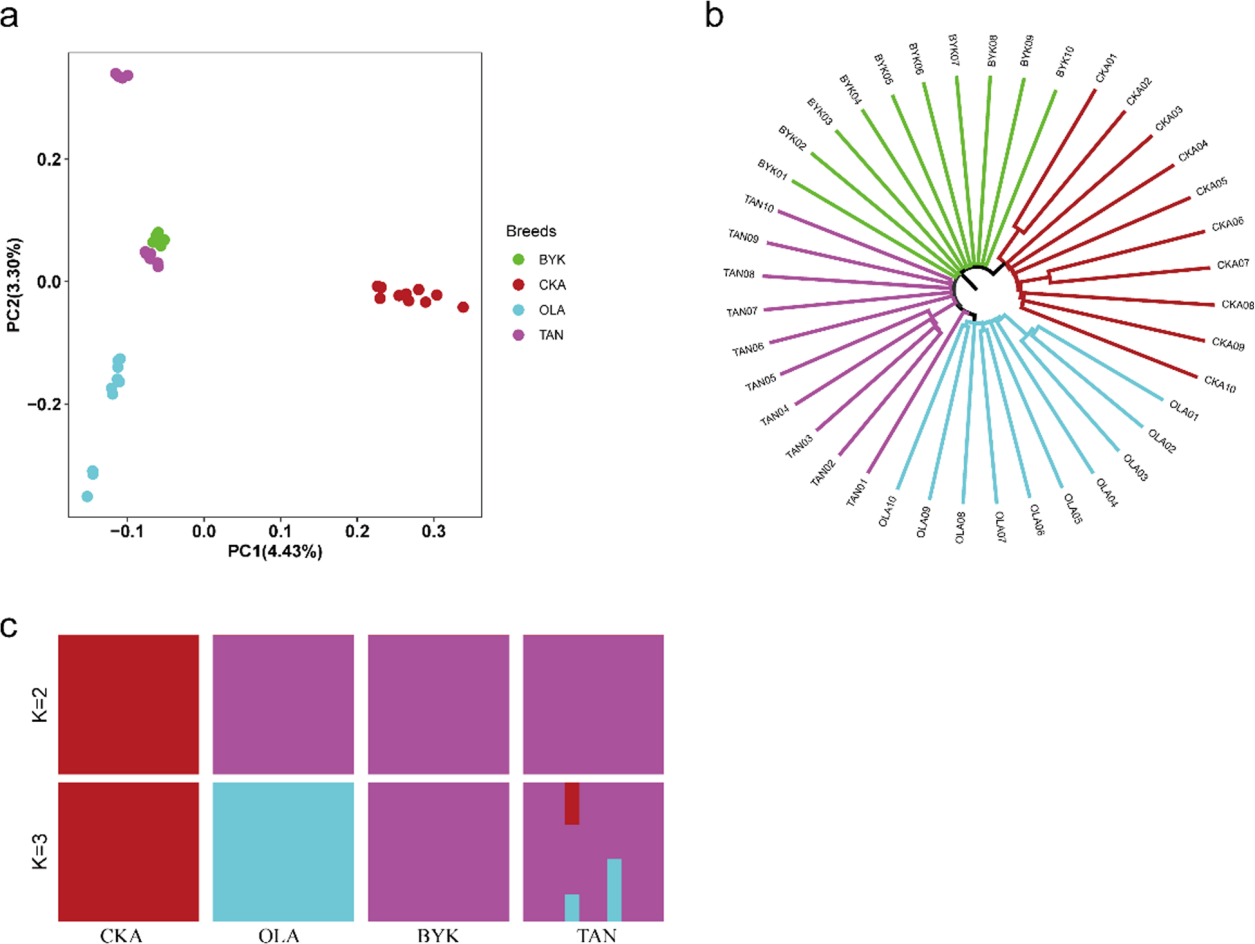
Analysis of the population structure in four sheep breeds. **a** Principal component analysis of 40 individuals. **b** Neighbor-joining tree of 40 individuals. **c** Bar plot of ancestry compositions by ADMIXTURE with the assumed number of ancestries (K = 2 and 3). CKA, Chaka sheep; OLA, Oula sheep; BYK, Bayinbuluke sheep and TAN, Tan sheep

Most of the sheep breeds included in this study originated from western China. The PCA showed that the first component (Fig. 2a) separated the CKA breed from the other breeds (Fig. 2a), explaining 4.43% of the total variance, which indicated a considerable genetic distance between the CKA breed and the other three breeds. Among these four breeds, OLA sheep were differentiated from the other breeds in the second component, explaining 3.30% of the total variance (Fig. 2a).

We explored the phylogenetic relationships using the autosomal genome data. The NJ tree demonstrated that CKA and OLA sheep grouped separately (Fig. 2b) while BYK and TAN sheep grouped in a single clade, consistent with PCA.

Clustering models were used for estimating ancestral populations with K = 2 and K = 3 by ADMIXTURE [11] (Fig. 2c). K changed progressively from 02 to 03 in the four analyzed breeds, providing evidence of admixture, the extent of which varied between the different breeds. The results suggested K = 2 as the most likely number of genetically distinct groups for our sample, reflecting the divergence of CKA from other breeds. At K = 3, TAN sheep represented clear evidence of genetic heterogeneity with shared genome ancestry with BYK sheep, which both belong to the Mongolian lineage.

### Specific SNPs and Indels in CKA sheep

**Fig. 3:**
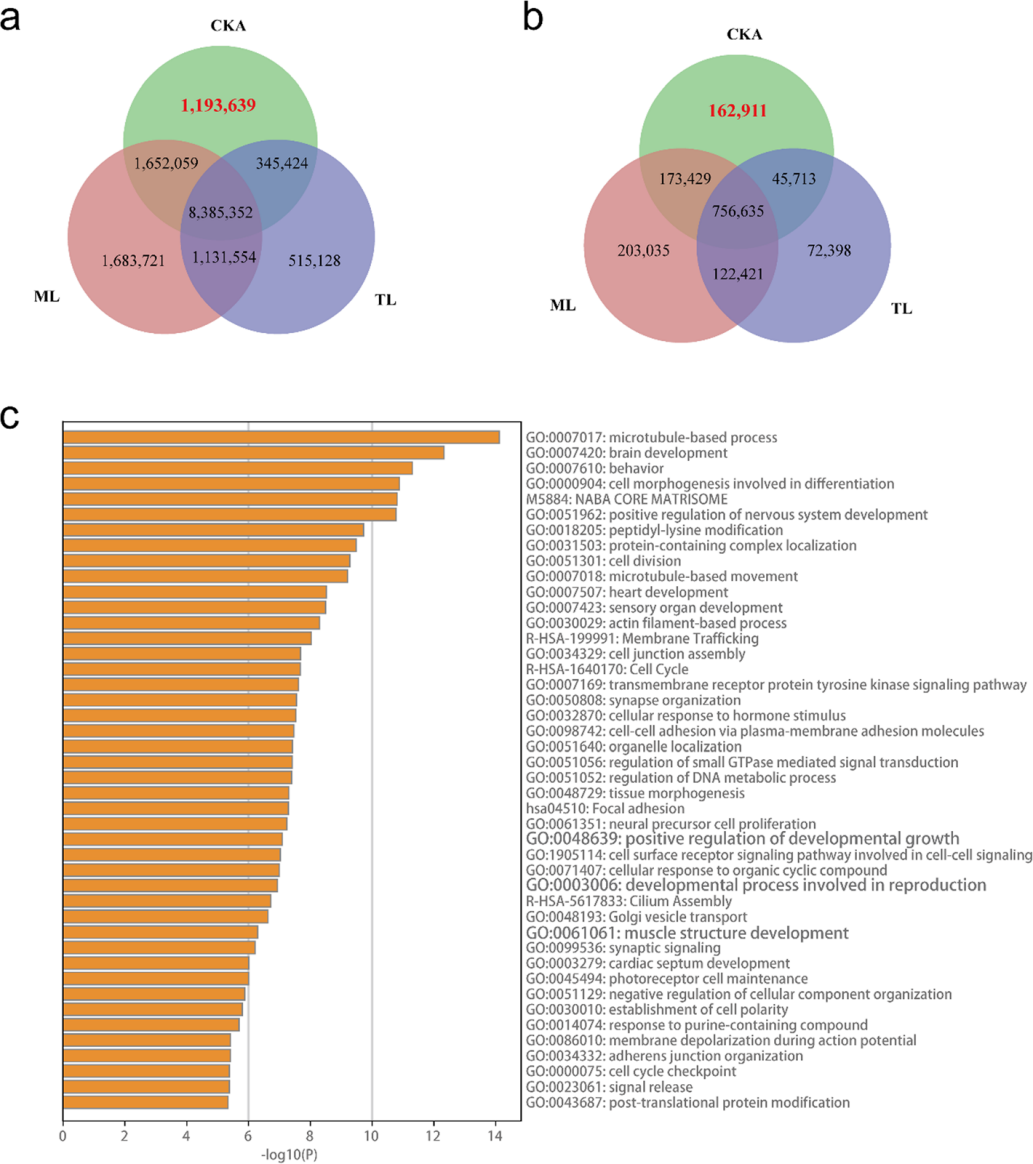
Specific SNP and indel in CKA sheep and the GO and KEGG analysis of its host gene. **a** The SNPs **b** The indel distribution in different breeds. **c** Heatmap for Top 44 GO and KEGG analysis of its host gene. CKA, Chaka sheep; ML, Mongolian lineage (TAN, Tan and BYK, Bayinbuluke sheep) and TL, Tibetan lineage (OLA, Oula sheep)

We detected 1,193,639 and 162,911 specific SNPs and indels in CKA sheep, respectively (Fig. 3a, b). Moreover, 2759 host genes were annotated by missense mutations, including SNPs and indels for Gene Ontology (GO) and Kyoto Encyclopedia of Genes and Genomes (KEGG) analysis. Finally, the genes annotated with missense mutations enriched muscle structure development (GO:0061061), the fibroblast growth factor receptor signaling pathway (GO:0008543), and skeletal myofibril assembly (GO:0014866) (Fig. 3c). Furthermore, 117 genes were identified to be associated with muscle structure development such as insulin-like growth factor 1 (*IGF1*), growth differentiation factor 3 (*GDF3*), histone deacetylase 9 (*HDAC9*), transforming growth factor beta receptor 2 (*TGFBR2*), and calpain 3 (*CAPN3*), among others. (Supplementary Table 4). Some genes also enriched nerve-related gene ontology shown in Fig. 3c.

### Genetic signature of positive selection in Chaka

**Fig. 4:**
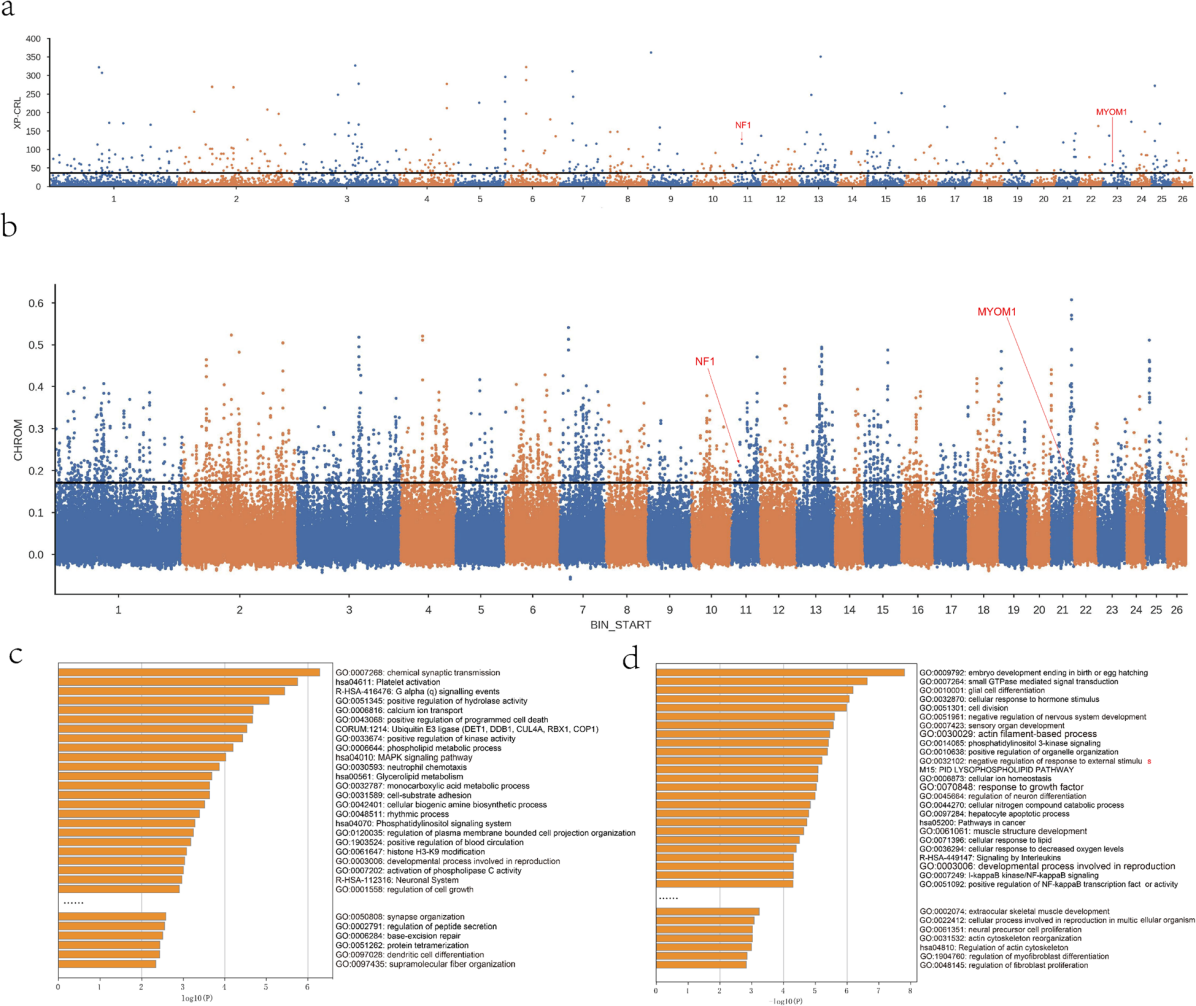
Genetic signature of positive selection in Chaka and GO and KEGG analysis. **a** The XP-CLR of CKA sheep based on other three sheep **b** The Fst on four breeds **c**. GO and KEGG heatmap selected analyzed by XP-CLR. **d** GO and KEGG heatmap selected analyzed by *F*st. The top 5% was chosen as the significant threshold for Fst and XP-CLR

The cross-population composite likelihood ratio (XP-CLR) was used to detect the selective signal by comparing CKA with other three breeds grouped together, and Fst detected positive selection signatures in CKA sheep compared with the other three sheep breeds as per the results of population structure (Fig. 4a, b).

In XP-CLR, 521 genes were selected for the downstream analyses (Fig. 4a and Supplementary Table 5). Notably, the genes were significantly enriched in the MAPK signaling pathway (hsa04010) and supramolecular fiber organization (GO:0097435) (Fig. 4c and Supplementary Table 6). The results suggested the enrichment of muscle-related genes including *MDS1* and *EVI1* complex locus (*MECOM*), Neurofibromin 1 (*NF1*), transforming growth factor beta 2 (*TGFB2*), phospholipase A2 group IVF (*PLA2G4F*), phosphatidylinositol-3,4,5-trisphosphate dependent Rac exchange factor 1 (*PRex1*) and myomesin 1 (*MYOM1*) which are crucial in the genome selection of CKA sheep.

Furthermore, 3619 genes showed a putatively high selection signature above the threshold by Fst (Fig. 4b and Supplementary Table 7). GO and KEGG analysis indicated its enrichment in muscle structure development (GO:0061061) (Fig. 4d and Supplementary Table 8). Furthermore, 62 genes were identified to be associated with the muscle structure development, such as 3-hydroxy-3-methylglutaryl-CoA reductase (*HMGCR*), lysine acetyltransferase 2A (*KAT2A*), RAR-related orphan receptor alpha (*RORA*), ubiquitin-specific peptidase 2 (*USP2*), and SRY-box transcription factor 6 (*SOX6*).

Lastly, *NF1* and *MYOM1* genes were positively selected in CKA sheep, indicating Fst and XP-CLR revealed adaptive and beneficial selection. In addition, some genes also enriched nerve-related genes, as shown in Fig. 4c and d.

## Discussion

CKA sheep are well known for their diverse alleles, more so than any of their commercial counterparts, and they have been a valuable genetic resource, potentially harboring unique gene pools resulting from long-term adaptation to the Chaka Salt Lake environment. To explore the genetic resources and to preserve the diverse gene pools of CKA sheep, we reported the genome-wide population structure, genetic diversity, and candidate signatures of positive selection in CKA sheep breeds for the first time using next-generation sequencing.

Combining the length of the ROH with LD decay, the CKA and TAN sheep were found to have higher genetic diversity than OLA and BYK sheep. F_ROH_ showed that CKA and TAN sheep have a lower inbreeding coefficient, which suggested their inbreeding depression was not severe. Since OLA sheep showed the highest inbreeding coefficient, definitive measures should be taken to prevent inbreeding depression. The estimated relative effective population size of the breeds 1000 years before present was OLA > TAN>CKA > BYK. Apart from the OLA sheep, the genetic diversity determined by effective population size was consistent with ROH and LD decay results (TAN>CKA > BYK). However, OLA sheep represented the largest effective population size, which may result from the fact that OLA sheep are within semi-feral populations that did not receive extensive human interventions [12].

The results of PCA, NJ-tree, and STRUCTURE suggest that the four sheep breeds could be subdivided into three genetic clusters: the CKA group, the Mongolian lineage group, and the Tibetan lineage group. In general, the partitioning of the breeds was consistent with their geographic distributions and breed properties. The PCA showed that CKA sheep completely separated from OLA, TAN, and BYK sheep. While OLA sheep, a famous Tibetan lineage breed, is completely separated from TAN and BYK. This result is consistent with previous reports [13]. For the NJ phylogenetic tree, the CKA and OLA sheep were clustered separately, while, BYK and TAN sheep clustered together. Furthermore, CKA sheep represented distinctive ancestry in the structure analysis. OLA sheep separated at K = 3, which shared its ancestry with TAN and BYK sheep (K = 2), a result also consistent with previous studies [13]. However, TAN sheep appeared to share some composition with CKA and OLA sheep, which requires further research.

Population structure analysis can distinguish geographical origins. In the current analysis, BYK and TAN sheep were observed to originate from Xinjiang and Ningxia provinces, respectively. Moreover, structural and phylogenetic analysis revealed that these breeds originated from the Mongolian lineage, possessing similar physiological characteristics. Besides, the Mongolian lineage group has a widespread distribution in China, which may be the result of Genghis Khan’s expedition during the Yuan dynasty, highlighting the superior adaptability and performance of these sheep populations [14]. The current data set provides strong evidence of the different population structures between CKA and the other three breeds. Further analyses are needed to develop an in-depth understanding of the relationships between the CKA sheep and Mongolian or Tibetan lineage groups.

Additionally, CKA sheep accounted for 8.25% (1,356,550/16,443,030) of total mutations. The specific missense mutations, including SNPs and indels of CKA sheep, annotated 2686 genes for functional annotation. The muscle structure development (GO:0061061) pathway was enriched by 117 genes, including *IGF1*, *GDF3*, *HDAC9*, *TGFBR2*, and *CAPN3*. The *IGF1* plays key roles in skeletal muscle physiology, including the promotion of essential protein synthesis for muscle repair or hypertrophic adaptation and the competition between cellular proliferation and differentiation [15]. *GDF3* is a regulator of myoblast proliferation, differentiation, and muscle regeneration [16]. *HDAC9* could suppress the transcriptional activity of MEF2 and thus impair the transcriptional circuitry of muscle differentiation through the negative-feedback loop [17]. The expression of the *CAPN3* gene is involved in progressive muscular dystrophies during early human development [18]. These gene functions determining muscle development help to provide a solid foundation for sheep breeding.

Various statistical tools such as Fst and XP-CLR are accepted tools in selection signature identification for livestock [19] as they can indicate the selective direction needed to identify a list of novel regions as potential selection targets. Meat quality is a quantitative trait regulated by complex factors such as glycolysis, stress reaction, cell cycle, proteolysis, protein ubiquitination, and apoptosis, among others [20]. GO and KEGG analysis indicated that the genes above the Fst threshold enriched muscle structure development (GO:0061061). Genes in selective signals identified by XP-CLR enriched the MAPK signaling pathway (hsa04010) and supramolecular fiber organization (GO:0097435). Combining Fst and XP-CLR, we were able to identify *NF1* and *MYOM1* genes positively selected for the development of muscles. *NF1* gene is required for skeletal muscle development [21] and essential for normal muscle function and survival [22]. Whereas, the *MYOM1* gene encodes a highly specific protein marker in cell differentiation of cross-striated muscle and might function in myofibril assembly and/or maintenance [23]. The subsarcolemmal cytoskeleton can be generally grouped into junctional and non-junctional domains [24]. For adult skeletal muscle fibers, the supramolecular organization of the subsarcolemmal cytoskeleton is also a crucial factor in influencing meat quality [25].

## Conclusion

The PCA, STRUCTURE, and NJ-tree analyses revealed that CKA sheep are distinct from BYK, TAN, and OLA sheep, and this breed has descended from a unique ancestor. The host genes annotated by specific missense mutations, including SNPs and indels of CKA sheep enriched muscle structure development (GO:0061061), which included *IGF1*, *GDF3*, *HDAC9*, *TGFBR2*, and *CAPN3*, genes, among others. Besides, strong selection signals were detected by Fst, which also enriched muscle structure development (GO:0061061). Strong selection by XP-CLR enriched MAPK signaling pathway (hsa04010) and supramolecular fiber organization (GO:0097435), identifying *NF1* and *MYOM1*, two common genes related to muscle development.

## Methods

### Ethics statement

This study was conducted according to the Faculty Animal Policy and Welfare Committee of Northwest A&F University (FAPWC-NWAFU).

### Sample collection and sequencing

We randomly selected CKA sheep (*n* = 10) for the study from 305 individuals scattered on the prairie [26]. Genomic DNA was extracted without euthanasia from 1 mL whole blood samples in 2% heparin [27]. DNA quality, purity, and concentration were detected by electrophoresis on 1% agarose gels, NanoPhotometer® spectrophotometry (IMPLEN, CA, USA), and the Qubit® DNA Assay Kit using the Qubit®2.0 Fluorometer (Life Technologies, CA, USA). After the purification of PCR products (AMPure XP system), the Agilent Bioanalyzer 2100 system was used to assess the library quality. Finally, the qualified libraries were subjected to HiSeq 4000 next-generation sequencing with paired-end mode. Trimmomatic (Leading:20 Trailing:20 Slidingwindow:3–15 Avgqual:20 Minlen:35 Tophred:33) was used to trim the sequencing reads, and FASTQC was used to assess the quality of the raw sequencing data. The qualified reads were then mapped, sorted, and deduped to the sheep reference genome (Oar_v4.0) by BWA-MEM (0.7.13-r1126) and Picard v2.18.2 (http://broadinstitute.github.io/picard/). The average sequencing depth was obtained for the 10 CKA sheep was 7.68×. The overall alignment rate and read coverage were 98.56 and 96.38%, respectively, across all samples. Detailed information about the mapping rate, duplication, insert size, mean depth, and amount of sequence was provided in Supplementary Table 9. Data for BAK (*n* = 10), TAN (*n* = 10), OLA (*n* = 10) sheep were downloaded from NCBI (SRR shown in Supplementary Table 10 with accession number SRP066883) [12]. Amongst these populations in China, CKA, TAN, BYK, and OLA sheep are widespread to central Qinghai (36.90°N, 98.46°E), Ningxia (37.78°N, 107.41°E), Xinjiang (43.03°N, 84.15°E), and eastern Qinghai (35.52°N, 102.02°E), respectively (Supplementary Figure 1 and Supplementary Table 10).

### Variant identification

To assess the genetic diversity of CKA sheep, the genomes of the four sheep breeds (Table 2) were assembled and genotyped using GATK (version 3.6–0-g89b7209) [28]. A total of 15,021,174 SNPs were retained after filtering for population structure and selection signature analyses.

**Table 1:** Average length, average number of ROH and average genomic inbreeding coefficient of four sheep groups

Breeds	Length of ROH (Mb)		Number of ROH		Inbreeding coefficient (F_ROH_)	
	Mean ± SE	Range	Mean ± SE	Range	Mean ± SE	Range
CKA	83.802 ± 33.817	43.370–143.466	146.9 ± 40.253	98–219	0.032 ± 0.0129	0.01658–0.05485
BYK	84.567 ± 21.996	65.722–142.802	160.2 ± 18.683	141–205	0.0323 ± 0.0084	0.02513–0.05460
OLA	104.600 ± 27.650	83.544–172.768	209.8 ± 27.531	178–270	0.04 ± 0.0106	0.03194–0.06606
TAN	69.003 ± 21.225	51.648–126.279	144.2 ± 22.803	114–192	0.0264 ± 0.0081	0.01975–0.04828

*CKA* Chaka sheep, *OLA* Oula sheep, *BYK* Bayinbuluke sheep, *TAN* Tan sheep

**Table 2:** Sample information about Chinese sheep breeds

Breeds	Sample size	Habitat	Altitude	Abbreviation
*Chaka sheep*	10	Qinghai	~ 4000	CKA
*Bayinbuluke sheep*	10	Xinjiang	2000–3500	BAK
*Tan sheep*	10	Ningxia	1000–2000	TAN
*Oula sheep*	10	Qinghai	~ 4000	OLA

Observed heterozygosity (Ho) and expected heterozygosity (He) were used as indicators of genetic diversity. The level of runs of homozygosity (ROH) was estimated using PLINK v1.9 [29–31] with the following parameters: chr-set 26 --maf 0.05 --homozyg-window-snp 50 --homozyg-snp 50 --homozyg-kb 300 --homozyg-density 50 --homozyg-gap 1000 --homozyg-window-missing 5 --homozyg-window-threshold 0.05 --homozyg-window-het 03. The inbreeding coefficient (F_ROH_) was calculated as the sum length of ROH divided by the full length of the reference genome (2,615,499,683 bp). Furthermore, linkage disequilibrium (LD) decay between pairwise SNPs was calculated by popLDdecay software [32]. The effective population size was estimated with a mutation rate of 1e-8 base per generation, minor allele frequency (MAF) < 0.2, and segment size was 2,000,000 and 100,000 iterations using PopSizeABC [33].

### Principal component analysis, phylogenetic tree, and STRUCTURE

The principal component analysis (PCA), phylogenetic tree, and STRUCTURE were analyzed using vcftools v0.1.12 (https://vcftools.github.io/index.html) to convert vcf into plink format. The minimum allele frequency (MAF) > 0.0057 was used to ensure that at least two alleles at each site in all groups were retained, while plink was used to remove the linkage sites in genomic data with parameters of “--indep-pairwise 50 5 0.2 “, and the filtered data were used for the subsequent analyses.

The PCA was conducted using EIGENSOFT (version 4.2), implementing the Tracy–Widom statistic to assess the significance of each principal component. PC1 and PC2 were plotted using the ggplot2 package in the R 3.6.1 software.

To establish the evolutionary relationship among the four sheep breeds, we constructed a phylogenetic tree with the neighboring (neighbor-joining, NJ) method using plink software with the matrix of pairwise genetic distances. The result was visualized by FigTree v1.4.4 (http://tree.bio.ed.ac.uk/software/figtree/).

To accurately identify the ancestral components of the four sheep breeds, the study used ADMIXTURE to estimate the ancestral composition of each individual with genome-wide unlinked sites. Optimization for population differentiation was conducted according to CV errors, and only two-three ancestors (K = 2/3) were used and visualized using R 3.6.1 (Supplementary Table 11). Because the results of K = 4 distorted the population differentiation, analysis with four ancestors (K = 4) was not conducted.

### Selection signatures

Interpopulation Wright’s Fst analyses were conducted between the four sheep breeds. Selection signature refers to genomic regions under artificial or natural selection, which are mainly characterized by certain regions of DNA fragment polymorphism, a more homozygous locus, and the increasing linkage disequilibrium extent. The fixation index (*F*_ST_) values [34] were calculated in sliding 50-kb windows with 20-kb steps along the autosomes using VCFtools [35]. The top 5% was chosen as the significance threshold for Fst.

The cross-population composite likelihood ratio (XP-CLR) is another method for the determination of the selection signal based on the differentiation of allele frequency across populations. It can avoid ascertainment biases and successfully detect older signals and the selections on standing variation [36]. The XP-CLR is a likelihood method for selection signature, which means the allele frequency changed very rapidly due to random drift between two populations [36]. Non-overlapping sliding windows of 50 kb were used, and the maximum number of SNPs within each window was 600. The top 5% was chosen as the significance threshold for XP-CLR.

### GO, KEGG pathway analysis

Annovar and Snpeff were deployed for gene annotations of ~ 15 million SNP, ~ 1.6 million indels [37, 38], and the selective signatures. Gene Ontology (GO) and Kyoto Encyclopedia of Genes and Genomes (KEGG) pathways were completed using metascape online software (http://metascape.org/gp/index.html#/main/step1) [39].

## Supplementary information

**Additional file 1: Supplementary Table 1.** The genetic variation information of SNPs and indels in sheep breeds. **Supplementary Table 2.** The level of genetic diversity in the four populations. **Supplementary Table 3.** Amount of ROH at different lengths. **Supplementary Table 4.** Host genes of CKA Specific missense mutations for GO and KEGG analysis. **Supplementary Table 5.** Summary of candidate genes in the Chaka sheep population detected by XP-CLR statistics. **Supplementary Table 6.** The list of GO and KEGG analyses for genes with high selection signature by XP-CLR. **Supplementary Table 7.** Summary of candidate genes in four sheep populations detected by Fst statistics. **Supplementary Table 8.** The list of GO and KEGG analyses for genes with high selection signature by Fst. **Supplementary Table 9.** The range and amount of sequence data. **Supplementary Table 10.** Sample collection and sequencing information. **Supplementary Table 11.** Optimum for population differentiation according to CV errors.

**Additional file 2:Supplementary Figure 1.** Geographic distribution of the four Chinese sheep breeds. (We state that the map and CKA images depicted in Supplementary Figure 1 were our own. TAN, BYK and OLA images were from this paper [12] and we obtained written permission from the copyright holders to use and adapt these images depicted in Supplementary Figure 1.)

## Data Availability

Raw sequence data of Tan, Bayinbuluke, and Oula sheep were downloaded from the National Center for Biotechnology Information (NCBI) Sequence Read Archive under accession number SRP066883 (https://www.ncbi.nlm.nih.gov/search/all/?term=SRP066883). Sequencing reads of Chaka sheep have been submitted to NCBI with accession number SRP237252 (https://www.ncbi.nlm.nih.gov/search/all/?term=SRP237252) (Supplementary Table 10). The sheep reference genome is Oar_v4.0 (https://www.ncbi.nlm.nih.gov/assembly/GCF_000298735.2/.)

## Abbreviations

Ho: Observed heterozygosity
He: Expected heterozygosity
ROH: Runs of homozygosity
LD: Linkage disequilibrium
SNPs: Single nucleotide polymorphisms
Indels: Insertion and deletion
TL: Tibetan lineage
ML: Mongolian lineage
CKA: Chaka
TAN: Tan
BYK: Bayinbuluke
OLA: Oula
GATK: Genome Analysis Toolki
MAF: Minimum allele frequency
PCA: Principal component analysis
NJ: Neighbor-joining
XP-CLR: Cross-population composite likelihood ratio
GO: Gene Ontology
KEGG: Kyoto Encyclopedia of Genes and Genomes
F_ROH_: Inbreeding coefficients
*IGF1*: Insulin-like growth factor 1
*GDF3*: Growth/differentiation factor 3
*HDAC9*: Histone deacetylase 9
*TGFBR2*: Transforming growth factor beta receptor 2
*CAPN3*: Calpain 3
*NF1*: Neurofibromin 1
*TGFB2*: Transforming growth factor beta 2
*PLA2G4F*: Phospholipase A2 group IVF
*PRex1*: Phosphatidylinositol-3,4,5-trisphosphate dependent Rac exchange factor 1
*MYOM1*: Myomesin 1
*HMGCR*: 3-hydroxy-3-methylglutaryl-CoA reductase
*KAT2A*: Lysine acetyltransferase 2A
*RORA*: RAR-related orphan receptor alpha
*USP2*: Ubiquitin specific peptidase 2
*SOX6*: SRY-box transcription factor 6
