# Supplementary material for: Population structure, genetic diversity, and selective signature of Chaka sheep revealed by whole genome sequencing
Source: BMC Genomics. 2020 Jul 29;21:520. doi: 10.1186/s12864-020-06925-z (PMC7391569; doi:10.1186/s12864-020-06925-z)
Supplement: Supplementary file 2 — Additional file 2:Supplementary Figure 1. Geographic distribution of the four Chinese sheep breeds. (We state that the map and CKA images depicted in Supplementary Figure 1 were our own. TAN, BYK and OLA images were from this paper [12] and we obtained written permission from the copyright holders to use and adapt these images depicted in Supplementary Figure 1.) [file 12864_2020_6925_MOESM2_ESM.docx]

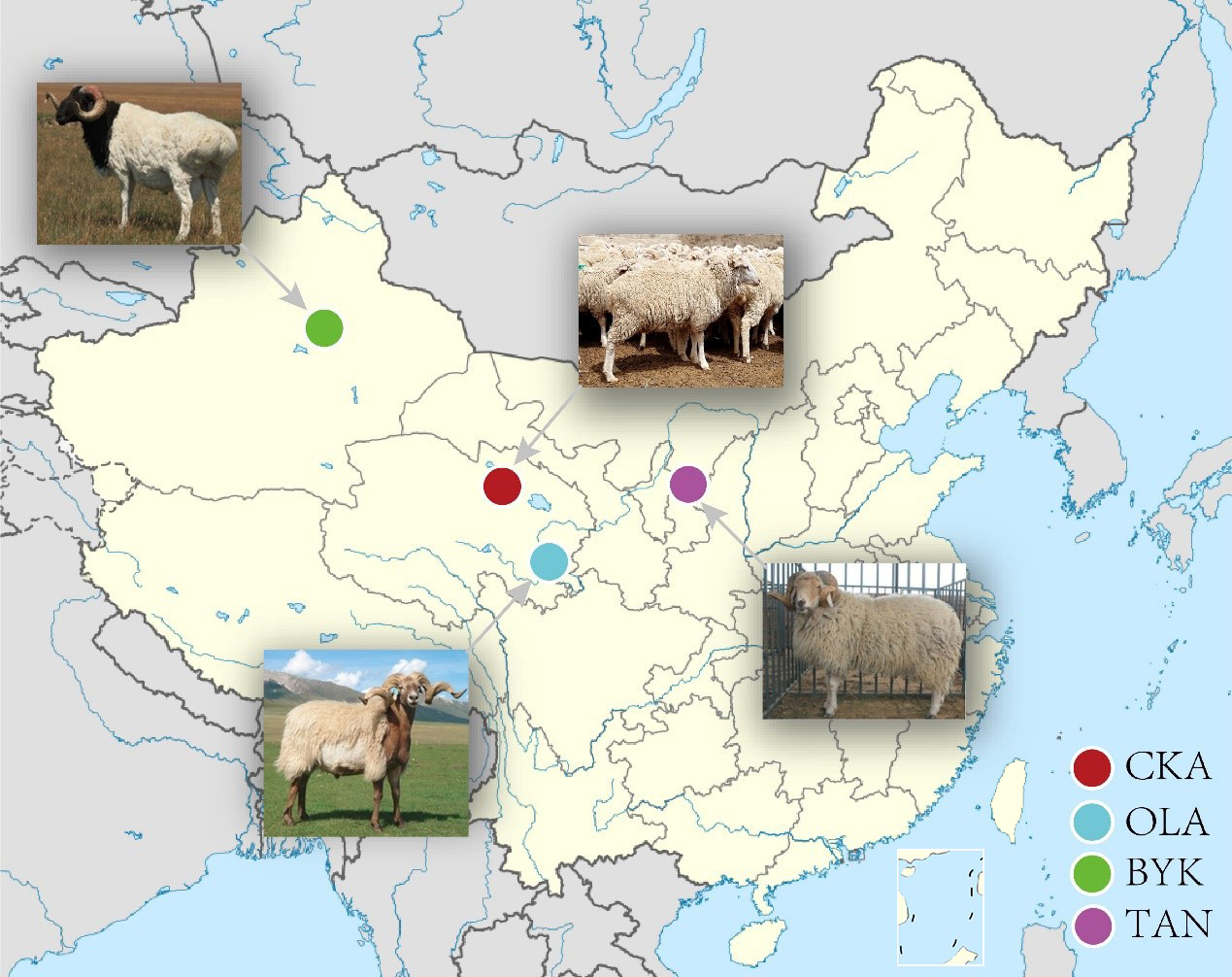


**Supplementary Fig. 1**. Geographic distribution of the four Chinese sheep breeds. (CKA, Chaka; TAN, Tan; BYK, Bayinbuluke and OLA, Oula sheep)
